# Trends in neoadjuvant chemotherapy use and oncological outcomes for muscle-invasive bladder cancer in Japan: a multicenter study

**DOI:** 10.18632/oncotarget.20991

**Published:** 2017-09-18

**Authors:** Go Anan, Shingo Hatakeyama, Naoki Fujita, Hiromichi Iwamura, Toshikazu Tanaka, Hayato Yamamoto, Yuki Tobisawa, Tohru Yoneyama, Takahiro Yoneyama, Yasuhiro Hashimoto, Takuya Koie, Hiroyuki Ito, Kazuaki Yoshikawa, Toshiaki Kawaguchi, Makoto Sato, Chikara Ohyama

**Affiliations:** ^1^ Department of Urology, Hirosaki University Graduate School of Medicine, Hirosaki, Japan; ^2^ Department of Urology, Tohoku Medical and Pharmaceutical University, Sendai, Japan; ^3^ Department of Advanced Transplant and Regenerative Medicine, Hirosaki University Graduate School of Medicine, Hirosaki, Japan; ^4^ Department of Urology, Aomori Rosai Hospital, Hachinohe, Japan; ^5^ Department of Urology, Mutsu General Hospital, Mutsu, Japan; ^6^ Department of Urology, Aomori Prefectural Central Hospital, Aomori, Japan

**Keywords:** bladder cancer, carboplatin, cisplatin, neoadjuvant chemotherapy, trends in use

## Abstract

**Objective:**

Despite benefits of neoadjuvant chemotherapy (NAC), the adoption of guideline recommendations for NAC use in patients with muscle-invasive bladder cancer (MIBC) has been slow. We aimed to evaluate temporal trends in NAC use and oncological outcomes in a representative cohort of patients with MIBC.

**Methods:**

We included 532 patients from 4 hospitals who underwent radical cystectomy (RC) for ≥ cT2 MIBC in 1996–2017. We retrospectively evaluated temporal changes in NAC use and progression-free and overall survival. Candidates for NAC were administered with either cisplatin- or carboplatin-based regimens. The impact of NAC on oncological outcomes was examined using multivariate Cox regression analysis with inverse probability of treatment weighting (IPTW) models.

**Results:**

Of 532 patients, 336 underwent NAC followed by RC (NAC group) and 196 underwent RC alone (Ctrl group). NAC use significantly increased from 10% (1996–2004) to 83% (2005–2016). The number of patients administered with cisplatin- and carboplatin-based regimens was 43 and 280, respectively. Oncological outcomes in the NAC group were significantly improved compared to those in the Ctrl group. Multivariable analysis with IPTW models revealed that NAC significantly improved oncological outcomes in patients with MIBC. A nomogram for 5-year overall survival predicted 16% improvement in patients undergoing NAC.

**Conclusions:**

NAC use for MIBC increased after 2005. Platinum-based NAC for MIBC potentially improves oncological outcomes.

## INTRODUCTION

Bladder cancer is the 11^th^ most commonly diagnosed cancer and the 14^th^ leading cause of cancer deaths worldwide [1]. Radical cystectomy (RC) with extended pelvic lymph node dissection is the standard treatment for non-metastatic muscle-invasive bladder cancer (MIBC) [2, 3]. Despite improvements in surgical and medical treatments, prognosis after RC has not improved over the past 2 decades. A multimodal approach including neoadjuvant chemotherapy (NAC) and surgical resection has been associated with improved survival in select patients undergoing RC [4, 5]. NAC use has been included in the contemporary guidelines on the management of MIBC [6, 7]. However, the adoption of NAC for MIBC has been slow and inconsistent. Several studies have suggested that only 1.4%–20.9% of patients with MIBC underwent NAC even in the contemporary series [8–10]. Several reasons have been proposed for the low utilization of NAC, including proportions of elderly patients with MIBC, poor performance status, multiple comorbidities, and impaired renal function. Although current guidelines recommend cisplatin-based NAC for patients with MIBC [11], approximately 40% of the patients are ineligible because of nephrotoxicity [12, 13]. No current data is available supporting the use of non-cisplatin-based regimens for patients with urothelial carcinoma who are unsuitable for neoadjuvant cisplatin treatment. Under difficult situations, carboplatin-based regimens are used as alternatives and are reportedly efficacious in the treatment of patients with renal impairment [13–17] or clinical T2 disease [18]. Our previous studies has suggested that a gemcitabine plus carboplatin (GCarbo) regimen with low toxicity facilitated the completion of neoadjuvant therapy without a dose reduction, prevented the delay in radical cystectomy, and resulted in a favorable oncological outcome [13, 17, 19]. However, studies regarding this data are limited [14, 20], and ideal regimens for NAC in patients with renal impairment remain unclear.

Currently, no information is available describing NAC use for patients with MIBC in Japan. Therefore, we investigated the trends in NAC use prior to RC and compared the oncological outcomes between patients administered with and without platinum-based NAC for MIBC in a multicenter setting.

## RESULTS

### Baseline characteristics

**Figure 1:**
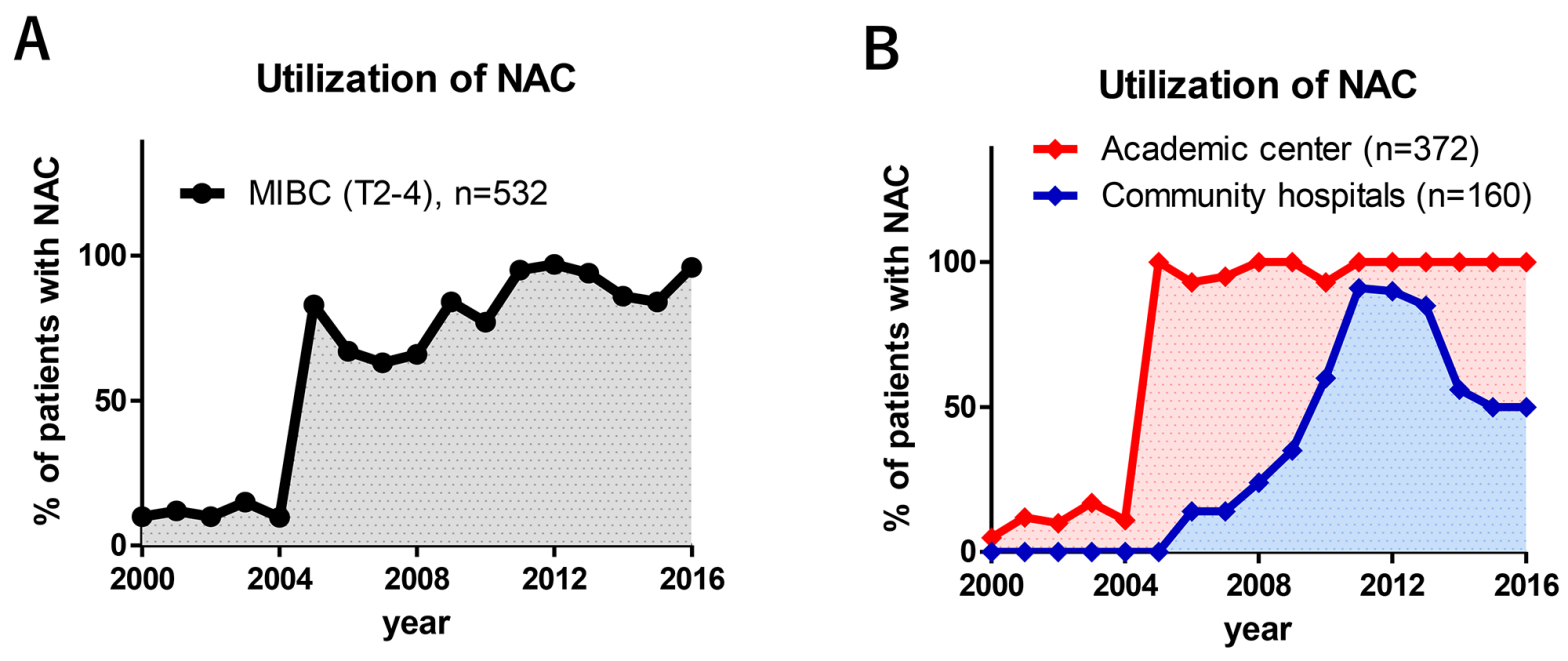
Trends in the use of neoadjuvant chemotherapy (NAC) NAC use increased from 9.7% in 2004 to 96% in 2016; its use was significantly increased after 2005. Median rate of NAC use before and after 2005 were 10% and 83%, respectively **(A)**. NAC use steadily increased between 2006 and 2011 in community hospitals, whereas it was promptly increased in the academic center in 2005 **(B)**.

In our cohort, 336 of 532 (63%) patients underwent NAC followed by RC (NAC group) and 196 of 532 (37%) underwent RC alone (Ctrl group). NAC use increased from 9.7% in 2004 to 96% in 2016. The median rate of NAC use before and after 2005 was 10% and 83%, respectively (Figure 1A, *P* < 0.001). After the beginning of NAC use at the academic medical center in 2005, NAC use in the community hospitals steadily increased between 2005 (0%) and 2011 (91%) (Figure 1B).

**Figure 2:**
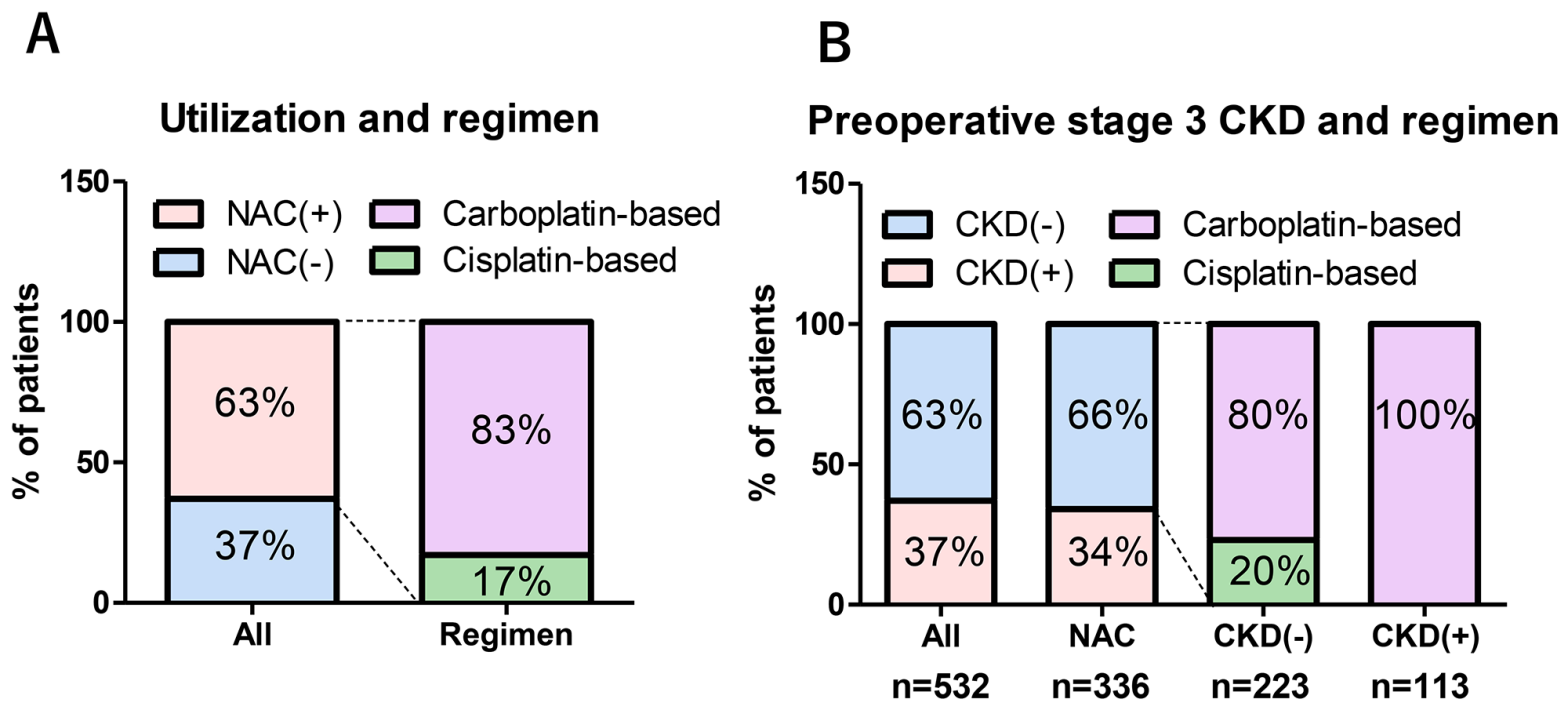
Prevalence of NAC use and the impact of chronic kidney disease (CKD) on regimens Of 532 patients, 336 (63%) received neoadjuvant chemotherapy (NAC) followed by radical cystectomy. Most patients in the NAC group received a carboplatin-based regimen (83%). Regimens in the NAC group were gemcitabine plus carboplatin (GCarbo) in 280 (83%), gemcitabine plus cisplatin (GCis) in 43 (13%), and others in 13 (3.9%) cases **(A)**. Of 532 patients, the number of those with and without preoperative stage 3 chronic kidney disease (CKD) was 195 (37%) and 337 (63%), respectively. Among patients with NAC, 223 of 336 (66%) did not have stage 3 CKD. Of 223 patients with NAC who did not have stage 3 CKD, 179 patients (80%) received carboplatin-based regimens **(B)**.

Except for age, there were no significant differences in preoperative patient characteristics between the Ctrl and NAC groups (Table 1). Most patients in the NAC group were administered with a carboplatin-based regimen (83%). The regimens in the NAC group were gemcitabine plus carboplatin (GCarbo) in 280 patients (83%), gemcitabine plus cisplatin (GCis) in 43 patients (13%), and others (MVAC; methotrexate, vinblastine, doxorubicin, and cisplatin or a docetaxel-based regimen) in 13 (3.9%) patients (Figure 2A). Of 532 patients, the number of patients presented with and without preoperative stage 3 chronic kidney disease (CKD) was 195 (37%) and 337 (63%), respectively. Among the patients with NAC, 223 of 336 (66%) did not present with stage 3 CKD. Of these 223 patients with NAC, who did not present with stage 3 CKD, 179 patients (80%) were administered with carboplatin-based regimens (Figure 2B). The number of patients treated in 1994-2004 and 2005-2016 were 134 (25%) and 398 (75%), respectively. The number of patients treated in 1994-2004 in the Ctrl and NAC groups were 120 (61%) and 14 (4.2%), respectively (*P* < 0.001). The number of patients who underwent postoperative chemotherapies for metastatic disease were significantly higher in the Ctrl group (n = 58, 30%) than in the those of NAC group (n = 61, 18%, *P* = 0.002).

**Table 1:** Background of patients

	Ctrl	NAC	*P value*
n	196	336	
Age, years	69±9.4	67±8.9	*0.044*
Male, n=	156 (80%)	263 (78%)	*0.719*
ECOG PS >0, n=	7 (3.6%)	8 (2.1%)	*0.260*
Hypertension (HTN), n=	68 (35%)	97 (29%)	*0.168*
Cardiovascular disease (CVD), n=	22 (11%)	40 (12%)	*0.813*
Diabetes mellitus (DM), n=	21 (11%)	48 (14%)	*0.223*
Preoperative stage 3 CKD (eGFR < 60 mL/min/1.73m^2^), n=	82 (42%)	113 (34%)	*0.058*
Clinical stage, n=			*0.291*
cT2	103 (53%)	153 (46%)	
cT3	79 (40%)	154 (46%)	
cT4	14 (7%)	29 (9%)	
cN+	11 (6%)	31 (9%)	*0.114*
Surgical outcomes, n=			
Urinary diversion (Neobladder)	95 (48%)	193 (57%)	*0.046*
Post-operative complications (any)	54 (28%)	83 (25%)	*0.469*
Pathological outcomes, n=			*<0.001*
pT0	9 (5%)	77 (23%)	
pT1	35 (18%)	66 (20%)	
pT2	67 (34%)	98 (29%)	
pT3	55 (28%)	68 (20%)	
pT4	30 (15%)	27 (8%)	
Tumor grade (High)	187 (95%)	326 (97%)	*0.360*
cT – pT (mean ± standard deviation)	0.2 ± 1.0	0.9 ± 1.2	*<0.001*
Number of patients with downstaging	61 (31%)	198 (59%)	*<0.001*
LVI+	95 (48%)	96 (29%)	*<0.001*
pN+	36 (18%)	50 (15%)	*0.418*

### Tumor responses

**Figure 3:**
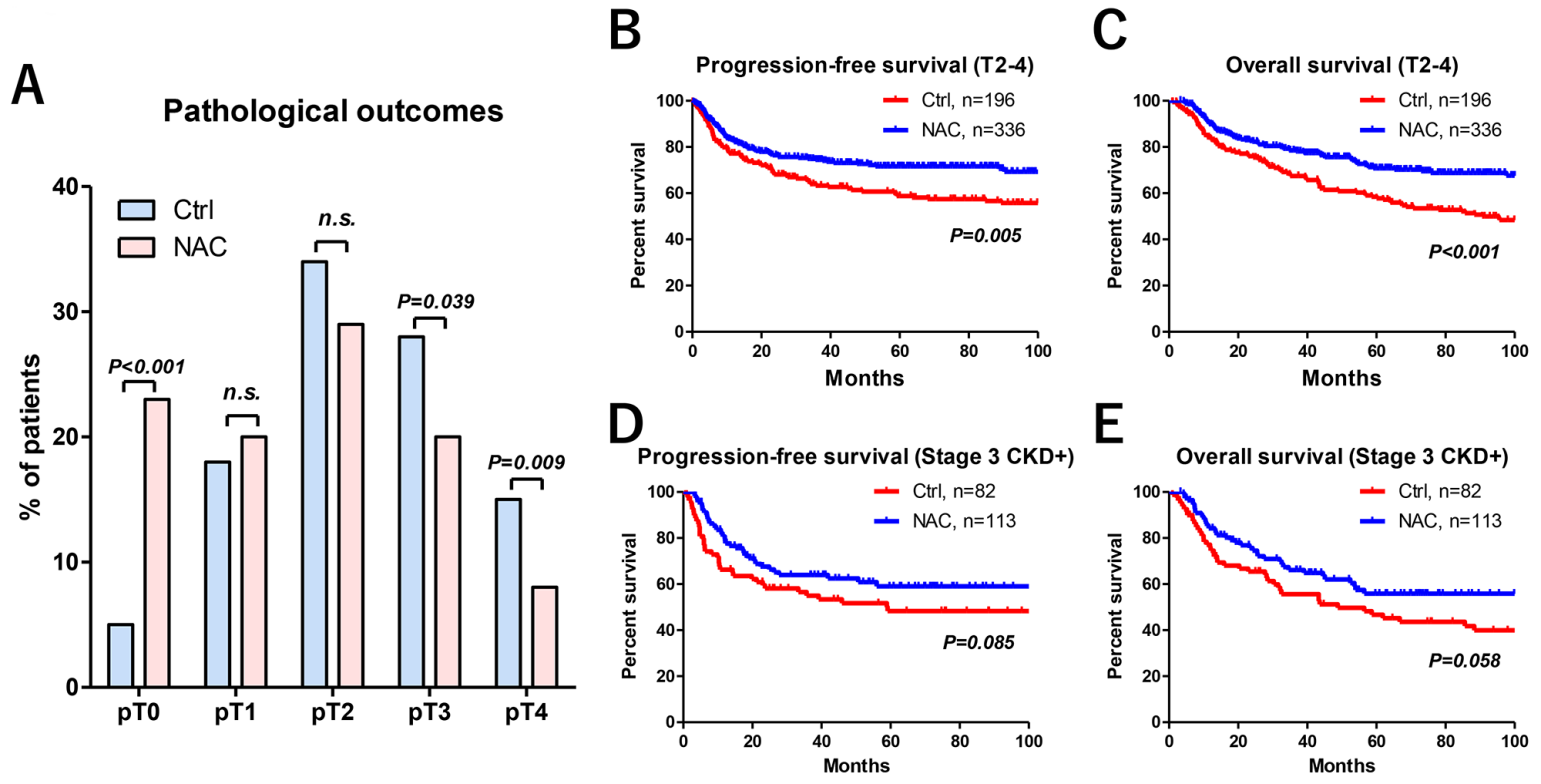
Pathological and oncological outcomes The number of pT0 and pT3-4 patients was significantly higher and lower in the NAC group than the Ctrl group, respectively **(A)**. In addition, pathological T0 was achieved in 23% of patients. There were significant differences in progression-free **(B)** and overall survival **(C)** measures between the Ctrl and NAC groups. No statistical difference was observed between groups in progression-free **(D)** and overall survival **(E)** in the patients with stage 3 CKD.

The number of pT3 and pT4 patients was significantly lower in the NAC group than that in the Ctrl group (Figure 3A). The mean pathological downstaging (cT–pT stage) in the primary tumor was significantly higher in the NAC group (0.9 ± 1.2) than that in the Ctrl group (0.2 ± 1.0) (*P* < 0.001) (Table 1). The number of patients who achieved pathological downstaging of the primary tumor was significantly higher in the NAC group (59%) than that in the Ctrl group (31%) (*P* < 0.001). In addition, the number of pT0 patients was significantly higher in the NAC group (n = 77, 23%) than that in the Ctrl group (n = 9, 5%, Figure 3A). Pathological T0 was achieved in 5.7% and 17% in cisplatin- and carboplatin-based regimens, respectively. The number of lymphovascular invasion (LVI)-positive patients was significantly lower in the NAC group (n = 96, 29%) than that in the Ctrl group (n = 95, 48%) (*P* < 0.001) (Table 1).

### Oncological outcomes

Median-follow-up periods in the Ctrl and NAC groups were 57 and 49 months, respectively. Progression-free survival (PFS) and overall survival (OS) measures between the Ctrl and NAC groups were statistically different (Figures 3B and 3C). The NAC group had significantly better 5-year PFS (72% vs 59%, *P* = 0.005) and 5-year OS (71% vs 58%, *P* < 0.001) than the Ctrl group. However, no difference was observed between the groups in terms of PFS (*P* = 0.085, Figure 3D) and OS (*P* = 0.058, Figure 3E) in the patients with stage 3 CKD.

**Figure 4:**
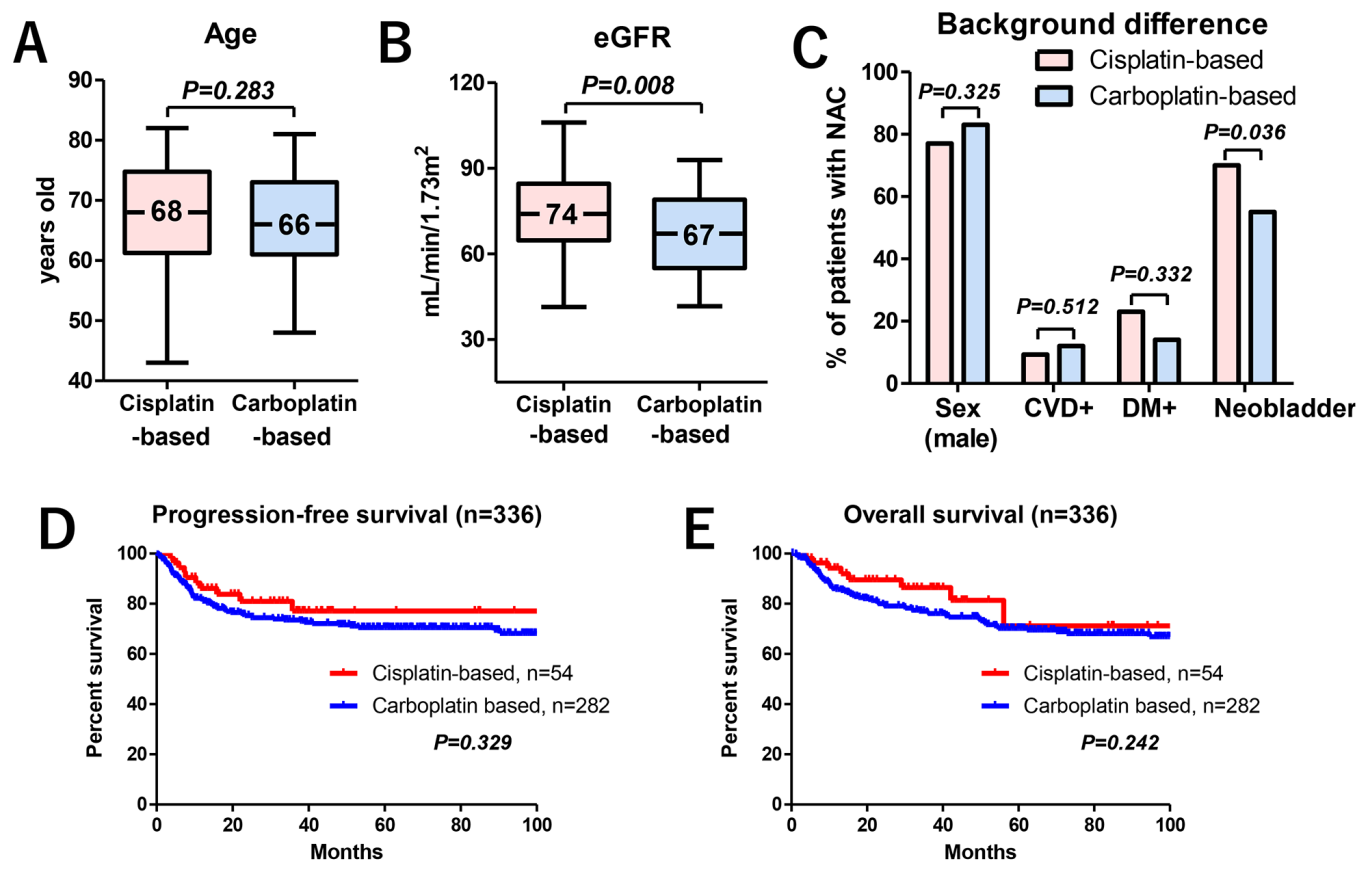
The impact of NAC regimens on oncological outcomes The median age for those given cisplatin- (68 years) or carboplatin-based regimens (66 years) was not statistically significant (P = 0.283) **(A)**. The median eGFR was significantly lower in NAC patients who received carboplatin-based therapy (67 ml/min/1.73 m^2^) than in those who received cisplatin-based therapy (74 ml/min/1.73 m^2^) **(B)**. No differences were seen in baseline characteristics between cisplatin- and carboplatin-based therapy including sex (77% vs 83%), CVD (9.3% vs 12%), and DM (23% vs 14%) except for an indication for orthotopic ileal neobladder substitution (70% vs 55%) **(C)**. There were no significant differences between PFS **(D)** and OS **(E)** when comparing the cisplatin- and carboplatin-based therapies.

Of the 336 patients who underwent NAC, the difference in median age of patients administered with cisplatin- (68 years, IQR: 61–75) or carboplatin-based regimens (66 years, IQR: 61–73) was not statistically significant (*P* = 0.283) (Figure 4A). The median courses of NAC were 2 in both regimens. Because of the patient selection for cisplatin eligibility [21], the median eGFRs in the patients undergoing carboplatin-based therapy were significantly lower than that in patients undergoing cisplatin-based therapy (67 vs 74 ml/min/1.73 m^2^, respectively) (Figure 4B). Except for an indication for orthotopic ileal neobladder substitution, no differences were observed in the baseline characteristics, such as sex, cardiovascular disease (CVD), and diabetes (DM), between the patients undergoing cisplatin- or carboplatin-based therapy (Figure 4C). There were no significant differences in PFS (Figure 4D) and OS (Figure 4E) measures between patients undergoing cisplatin- or carboplatin-based therapy.

### Uni- and multivariate analyses for prognosis

In univariate and multivariate Cox regression analysis, NAC was selected as an independent factor for PFS and OS (Table 2). Multivariate Cox regression analyses with an inverse probability of treatment weighting (IPTW) model revealed that the impact of NAC on PFS (*P* = 0.003; HR, 0.63) and OS (*P* < 0.001; HR, 0.56) was significant (Table 2). Univariate analyses revealed that the impact of NAC on PFS and OS was not significant in patients with stage 3 CKD (Table 3, upper row). However, multivariate analyses with an IPTW model revealed that the impact of NAC on PFS (*P* = 0.009; HR, 0.54) and OS (*P* = 0.023; HR, 0.59) was significant in patients with stage 3 CKD (Table 3, lower row).

**Table 2:** Cox regression analysis for prognosis for all patients (n=532)

Univariate	Factor	*P value*	HR	95%CI
Progression-free survival	Age (continuous)	*0.042*	1.02	1.00-1.04
	Sex (male)	*0.829*	0.96	0.67-1.38
	ECOG PS (continuous)	*0.623*	1.23	0.54-2.77
	CVD (positive)	*<0.001*	2.25	1.53-3.30
	DM (positive)	*0.847*	0.96	0.60-1.52
	Preoperative stage 3 CKD	*<0.001*	1.80	1.33-2.44
	NAC (underwent)	*0.003*	0.63	0.47-0.86
	cT3-4	*<0.001*	2.08	1.52-2.86
	cN+	*<0.001*	2.05	1.56-2.70
	Indication for neobladder	*<0.001*	0.44	0.32-0.60
Overall survival	Age (continuous)	*<0.001*	1.04	1.02-1.06
	Sex (male)	*0.697*	0.93	0.66-1.32
	ECOG PS (continuous)	*0.538*	1.30	0.57-2.96
	CVD (positive)	*<0.001*	2.09	1.42-3.06
	DM (positive)	*0.808*	1.06	0.68-1.64
	Preoperative stage 3 CKD	*<0.001*	1.96	1.46-2.63
	NAC (underwent)	*<0.001*	0.56	0.42-0.75
	cT3-4	*0.002*	1.61	1.20-2.16
	cN+	*0.016*	1.48	1.08-2.05
	Indication for neobladder	*<0.001*	0.47	0.35-0.64

Multivariate	Factor	*P value*	HR	95%CI
Progression-free survival	Age (continuous)	*0.523*	1.01	0.99-1.03
	Sex (male)	*0.697*	0.93	0.64-1.35
	ECOG PS (continuous)	*0.705*	0.85	0.36-2.01
	CVD (positive)	*<0.001*	2.43	1.64-3.62
	DM (positive)	*0.820*	0.95	0.59-1.52
	Preoperative stage 3 CKD	*0.016*	1.48	1.08-2.03
	NAC (underwent)	*0.001*	0.58	0.43-0.80
	cT3-4	*<0.001*	1.83	1.31-2.55
	cN+	*<0.001*	2.02	1.49-2.73
	Indication for neobladder	*<0.001*	0.54	0.39-0.75
Overall survival	Age (continuous)	*0.003*	1.03	1.01-1.05
	Sex (male)	*0.822*	0.96	0.68-1.37
	ECOG PS (continuous)	*0.409*	0.69	0.29-1.67
	CVD (positive)	*<0.001*	2.10	1.42-3.11
	DM (positive)	*0.875*	1.04	0.66-1.62
	Preoperative stage 3 CKD	*0.006*	1.54	1.13-2.09
	NAC (underwent)	*<0.001*	0.59	0.43-0.79
	cT3-4	*0.013*	1.48	1.09-2.02
	cN+	*0.005*	1.65	1.17-2.33
	Indication for neobladder	*0.002*	0.62	0.46-0.85

Multivariate (IPTW* model)	Factor	*P value*	HR	95%CI
Progression-free survival	NAC (underwent)	*0.003*	0.63	0.47-0.86
Overall survival	NAC (underwent)	*<0.001*	0.56	0.42-0.75

*, IPTW: inverse probability of treatment weighting. Adjusted variables: age, sex, ECOG PS, preoperative stage 3 CKD, HTN, DM, CVD, cT, cN, High grade, and indication for neobladder. Preoperative stage 3 CKD: eGFR <60 mL/min/1.73m^2^

**Table 3:** Cox regression analysis for prognosis for patients with stage 3 CKD (n=195)

Univariate	Factor	*P value*	HR	95%CI
Progression-free survival	NAC (underwent)	*0.074*	0.66	0.42-1.04
Overall survival	NAC (underwent)	*0.060*	0.66	0.43-1.02

Multivariate (IPTW model)	Factor	*P value*	HR	95%CI
Progression-free survival	NAC (underwent)	*0.009*	0.54	0.41-1.10
Overall survival	NAC (underwent)	*0.023*	0.59	0.38-0.93

IPTW: inverse probability of treatment weighting. Adjusted variables: age, sex, ECOG PS, HTN, DM, CVD, cT, cN, High grade, and indication for neobladder

### The nomogram for 5-year OS probability

**Figure 5:**
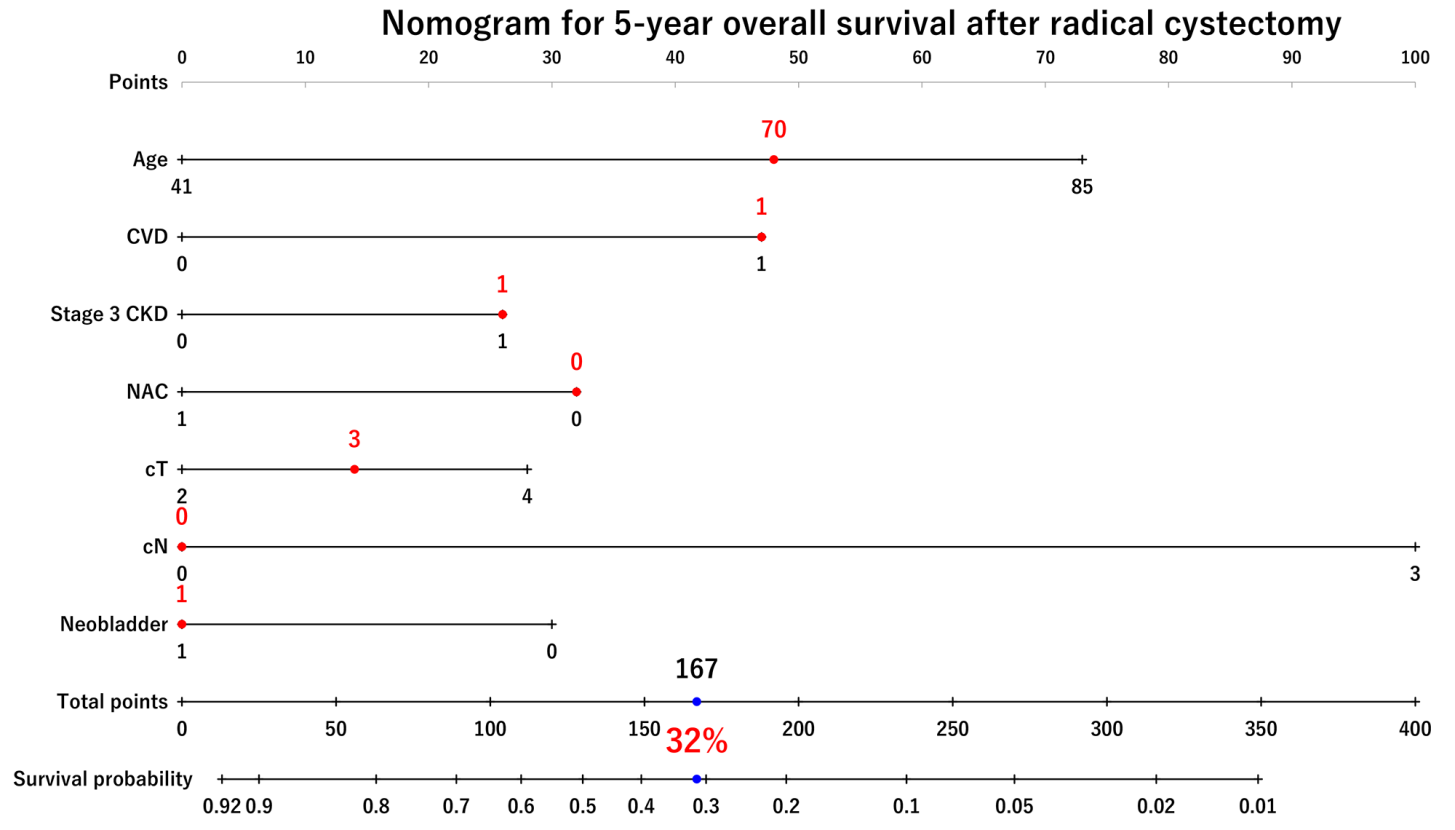
Nomogram for 5-year overall survival probability The nomogram predicting 5-year OS showed that NAC use improved the 5-year risk of OS from 32% to 48% (a 16% improvement) in 70-year-old patients with CVD+, stage 3 CKD, cT3, cN-, and indication for orthotopic ileal neobladder substitution.

We developed a nomogram to predict 5-year OS including preoperative factors, such as age, CVD, preoperative stage 3 CKD, NAC, cT, cN, and urinary diversion (Figure 5). Preoperative factors included in the nomogram were selected using multivariate Cox regression analysis (Table 2). NAC use improved the 5-year risk of OS from 32% to 48% (a 16% improvement) in 70-year-old patients with CVD+, stage 3 CKD, cT3, cN−, and an indication for orthotopic ileal neobladder substitution. The risk calculations for OS are provided in the Supplementary File (MS Excel, Supplementary File 1).

## DISCUSSION

The essential findings of the present study were that NAC use for patients with MIBC at our medical center has considerably increased over the last decade and has potential to improve oncological outcomes despite carboplatin-based NAC use. Multivariate Cox regression analysis with an IPTW model revealed that the impact of NAC on PFS and OS was significant. A nomogram for 5-year OS predicted 16% improvement by adding NAC in a relatively common patient, such as 70-year-old patients with CVD+, stage 3 CKD, cT3, cN−, and an indication for orthotopic ileal neobladder substitution. To our knowledge, the present study is the largest study evaluating the trends NAC use and oncological outcomes in patients with MIBC in Japan.

Although the body of evidence has suggested a survival benefit of NAC use for MIBC [5, 22], historical use of a NAC paradigm has been remarkably low. NAC use tends to be concentrated only in high-volume hospital and/or academic medical centers. However, a recent review that evaluated the recent trends in NAC use has suggested small but progressively increased use, which is approximately 20% of RC [8]. Zaid et al. have reported that the trend of NAC use increased from 10.2% in 2006 to 20.9% in 2010 in 5692 patients with MIBC in the National Cancer Database [23]. In Asia, Korean groups have reported a significant increase in NAC use from 4.6% between 2003 and 2005 to 8.4% between 2010 and 2013 in 1324 patients with MIBC [9]. The structural difficulties that have been reported to prevent NAC use are old age, comorbidities, physician preference, delayed RC, geographical region, and socioeconomic status of patients [8]. In the present study, we revealed a remarkably higher rate of NAC use (83% between 2005 and 2016). The reasons for this higher rate may be the inclusion of cisplatin-ineligible patients for a carboplatin-based regimen administration, universal health insurance coverage for the entire Japanese population, and short-term NAC followed by immediate RC. In Japan, urologists can prescribe NAC and plan RC as a part of sequential therapy in the urology ward. Consequently, we can optimize the treatment schedules without delay. Our results suggest that short-term NAC followed by immediate RC may not impede the patient outcomes [13, 15, 18, 19]. However, our results could not apply to other nations under different medical systems because of regional bias in Japan. The other possible reason is the achievement of a medical partnership between the academic medical center and community hospital in our urological alliance. All young urologists rotate between the academic medical center and the community hospital every 6–12 months in our urological educational program. They circulated the NAC paradigm to community hospitals after 2005, which resulted in a rapid increase in NAC use (0% to 91% in 5 years). Therefore, the development of people-to-people links within the educational programs might be one of the key factors for significantly increasing NAC use in our area.

Our retrospective evaluation of post-therapy pathological outcomes and prognosis showed feasibility and potential efficacy of NAC for MIBC, which was comparable to the results of the previous randomized trials and meta-analysis [4, 5, 24]. The number of patients who achieved pathological downstaging of the primary tumor was significantly higher in patients undergoing NAC (59%) than that in patients not undergoing NAC (31%). In addition, pathological T0 was achieved in 23% of patients (5.7% and 17% in cisplatin- and carboplatin-based regimens, respectively). Lymphovascular invasion, which is a strong prognostic factor for relapse [25–27], in the NAC group (29%) was significantly decreased compared to that in the Ctrl group (48%). NAC for MIBC significantly prolonged PFS (*P* = 0.003; HR, 0.63; 95% CI, 0.47−0.86) and OS (*P* < 0.001; HR, 0.56; 95% CI, 0.42−0.75) even though most patients were administered with carboplatin-based regimens. Although the impact of NAC on prognosis might be limited because of the retrospective nature of our study, our results support the clinical benefits of NAC for MIBC.

The use of carboplatin in a neoadjuvant setting and the optimal number of courses are still controversial [13–15, 18, 19, 28]. Because there is no evidence that clearly supports the superiority of a cisplatin-based regimen over a carboplatin-based regimen in a neoadjuvant setting for MIBC [15, 29], we designed a strategy including carboplatin-based NAC followed by RC in patients who had impaired renal function. Because multiple studies have suggested that a >90 days delay in undergoing RC is associated with adverse oncological outcomes [30–32], we performed 2 courses of NAC followed by RC within the 90 days by urologists. In our cohort, only 6 patients (1.8%) received ≥3 courses of NAC. Although the inclusion of cisplatin-ineligible patients for carboplatin-based NAC needs to be studied, our previous study has reported the efficacy and safety of carboplatin-based NAC followed by RC in patients with MIBC [13, 15, 18, 19]. The present study also showed that there were no clear differences in the prognosis between the two regimens. However, clinical benefits among preoperative stage 3 CKD patients might be limited compared with those in non-CKD patients with MIBC. As shown in Figures 3D and 3E, PFS (*P* = 0.085) and OS (*P* = 0.058) showed a marginal, but not a significant, difference between the Ctrl and NAC groups. Several studies have suggested that carboplatin-based NAC might be insufficient because of poor oncological outcomes in patients with MIBC [4, 22, 24]. The precise mechanism between renal impairment and cancer progression remains unclear and treatment in these patients is challenging. Although the inclusion of cisplatin-ineligible patients for carboplatin-based NAC invites debate, background adjusted multivariate Cox regression analysis with an IPTW model revealed that the impact of NAC on PFS and OS was significant. Although it is difficult to draw a definitive conclusion regarding the efficacy of a carboplatin-based NAC from the present study, it is worth noting the potential activity of a carboplatin-based regimen because it could be a viable option in patients with MIBC who are ineligible for cisplatin-based therapy.

Several limitations need to be acknowledged including the limited sample size and retrospective study design. We were unable to control selection bias and other unmeasurable confounders despite the use of statistical methods. Due to the retrospective nature, we could not obtain the safety profiles in the NAC patients. Although we included cN+ in the present study, indications of NAC for cN+ patients remain unclear. We could not exclude the influence of the improvement of therapies for long-term study periods. In addition, optimal number of NAC cycles for MIBC remain undetermined. It is difficult to draw a definitive conclusion regarding the clinical benefit of carboplatin-based NAC due to the limited number of patients with selection bias. Furthermore, our results cannot be applied to other nations because of universal health insurance. Any evidences from randomized prospective trials are needed to determine best NAC regimens and cycles for MIBC in Japanese patients. Regardless of these limitations, this is the largest study to evaluate the trends in the use of NAC in Japan, and our study supports the potential benefit of NAC for MIBC.

In conclusion, trends in the use of NAC increased from 10% before 2004 to 83% between 2005 and 2016. The platinum-based short-term NAC followed by immediate RC for MIBC potentially improves oncological outcomes. Estimated 5-year OS improvement achieved 16%. A carboplatin-based regimen might be a useful alternative in MIBC patients who are ineligible for cisplatin. Further prospective randomized studies are needed to confirm the role of NAC in patients with MIBC.

## MATERIALS AND METHODS

### Design and ethics statement

This study was designed as a retrospective multi-center study and performed in accordance with the ethical standards of the Declaration of Helsinki and approved by an ethics review board of Hirosaki University School of Medicine (authorization numbers; 2016–225).

### Patient selection

Between May 1996 and February 2017, we performed RC in 581 consecutive patients with MIBC. The indications for NAC were locally advanced MIBC without distant metastasis, including cT2–4 or local lymph node involvement. Of 581 patients, 532 met the inclusion criteria. We stratified the patients into 2 groups, those who received NAC (NAC group) and those who had surgery alone (Ctrl group).

### Evaluation of variables

The variables analyzed were age, sex, body mass index, Eastern Cooperative Oncology Group performance status (ECOG PS), history of CVD, hypertension, DM, NAC regimen, clinical stage, tumor recurrence, and renal function. Renal function was evaluated using estimated glomerular filtration rate (eGFR) before RC by a modified version of the abbreviated Modification of Diet in Renal Disease Study formula for Japanese patients [33]. Stage 3 CKD was defined when preoperative eGFR < 60 mL/min/1.73 m^2^ persisted for >3 months. Tumor stage and grade were assigned according to the 2009 TNM classification of the Union for International Cancer Control [34]. Postoperative complications were evaluated by Clavien-Dindo classification [35]. PFS and OS were defined from the day of first treatment to the date of event onset.

### Neoadjuvant chemotherapy (NAC)

A regimen was selected based on the guidelines regarding eligibility for the proper use of cisplatin [21] and the patient’s overall status. Before September 2013, we mainly used GCarbo for NAC. Thereafter, we used gemcitabine plus cisplatin (GCis) for cisplatin-eligible patients. Some patients underwent a standard dose of methotrexate, vinblastine, adriamycin, and cisplatin (MVAC). All patients underwent chemotherapy in the hospital. Patients received either gemcitabine 800–1000 mg/m^2^ on days 1, 8, and 15 plus cisplatin 70 mg/m^2^ on day 2 every 3 weeks or gemcitabine 800–1000 mg/m^2^ on days 1, 8, and 15 plus carboplatin at an area under the curve of 4–4.5 according to the Calvert formula on day 2 every 3 weeks, for 2 to 4 cycles [16, 18]. Tumor response was evaluated after the second course of NAC. To reduce the delay of surgery, we planned 2 courses of NAC and surgery within 90 days [30]. Patients with insufficient tumor response (progressive disease) received 3 or 4 cycles of NAC.

### Surgical procedure

All patients underwent RC, urinary diversion, and standard pelvic lymph node dissection (PLND) using the basic technique we have described previously [36]. PLND included removal of the obturator, external iliac, hypogastric, and common iliac lymph node chains (there were no para-aortic or paracaval dissections). Orthotopic ileal neobladder construction, ileal conduit diversion, or cutaneous ureterostomy were performed according to previously reported methods [37–40].

### Patient follow-up

After treatment, each patient was assessed every 3–6 months with blood and serum tests, ultrasonography, and computed tomography (CT) for the detection of tumor recurrence. Adjuvant chemotherapy was not administered routinely. Salvage therapy was indicated when recurrent disease was detected by CT.

### Outcome evaluations

We retrospectively evaluated pathological T and N stages, post-therapy pathological downstaging (cT–pT stage), and LVI in the Ctrl and NAC groups. Oncologic outcomes for both groups, including PFS and OS, were investigated using the Kaplan–Meier method and compared with the log-rank test. In addition, we analyzed the impact of NAC on oncological outcomes among patients with stage 3 CKD. The impact of NAC regimens on oncological outcomes was investigated between the cisplatin- and carboplatin-based therapies. Multivariate Cox regression analysis was performed for independent predictors of PFS and OS.

### Statistical analysis

Statistical analyses of the clinical data were performed using SPSS version 24.0 (SPSS Inc., Chicago, IL, USA), GraphPad Prism 5.03 (GraphPad Software, San Diego, CA, USA), and R 3.3.2 (The R Foundation for Statistical Computing, Vienna, Austria). Categorical variables were compared using the Fisher exact test or Chi-squared test. Quantitative variables were expressed as mean with standard deviation (SD) or median with interquartile range (IQR). The statistical difference between groups was compared using the Student *t*-test for a normal distribution or the Mann–Whitney *U* test for an abnormal distribution. *P* values < 0.05 were considered statistically significant. Multivariate analyses with the Cox regression model and hazard ratio (HR) with 95% confidence interval (CI) were calculated. We also performed inverse probability of treatment weighted Cox regression analysis to evaluate the impact of NAC on prognosis. An IPTW method reweights both exposed and unexposed groups to emulate a propensity score-matched population [41]. Variables included in the IPTW model were age, sex, ECOG PS, HTN, CVD, DM, stage 3 CKD, cT, cN, tumor grade, and urinary diversion. We developed a prognostic factor-based risk stratification nomogram for 5-year OS with Cox proportional hazards regression analysis using the “rms” library in R.

## SUPPLEMENTARY MATERIALS





## Abbreviations

RC: radical cystectomy
MIBC: muscle invasive bladder cancer
IPTW: inverse probability of treatment weighting
ECOG PS: Eastern Cooperative Oncology Group Performance Status
CVD: cardiovascular disease
HTN: hypertension
DM: diabetes mellitus
eGFR: estimated glomerular filtration rate
CKD: chronic kidney disease
NAC: neoadjuvant chemotherapy
GCis: gemcitabine plus cisplatin
GCarbo: gemcitabine plus carboplatin
MVAC: methotrexate, vinblastine, adriamycin, and cisplatin
LVI: lymphovascular invasion
HR: hazard ratio
CI: confidence interval
SD: standard deviation
PFS: progression-free survival
OS: overall survival
CT: computed tomography
PLND: pelvic lymph node dissection
