# Supplementary material for: Trends in neoadjuvant chemotherapy use and oncological outcomes for muscle-invasive bladder cancer in Japan: a multicenter study
Source: Oncotarget. 2017 Sep 18;8(49):86130–42. doi: 10.18632/oncotarget.20991 (PMC5689672; doi:10.18632/oncotarget.20991)
Supplement: Supplementary file 1 [file oncotarget-08-86130-s001.pdf]

Trends in neoadjuvant chemotherapy use and oncological outcomes for muscle-invasive bladder cancer in Japan: a multicenter study

SUPPLEMENTARY MATERIALS

Supplementary File 1: Nomogram for 5-year overall survival probability. The impact of independent risk factors for 5-year overall survival (OS) in patients with muscle invasive bladder cancer (MIBC) after radical cystectomy.

See Supplementary File 1
